# Examining the role of the photopigment melanopsin in the striatal dopamine response to light

**DOI:** 10.3389/fnsys.2025.1568878

**Published:** 2025-04-02

**Authors:** L. Sofia Gonzalez, Austen A. Fisher, Kassidy E. Grover, J. Elliott Robinson

**Affiliations:** ^1^Division of Experimental Hematology and Cancer Biology, Department of Pediatrics, Cincinnati Children’s Hospital Medical Center, Cincinnati, OH, United States; ^2^Department of Pediatrics, University of Cincinnati College of Medicine, Cincinnati, OH, United States; ^3^Neuroscience Graduate Program, University of Cincinnati College of Medicine, Cincinnati, OH, United States

**Keywords:** dopamine, vision, melanopsin, fiber photometry, light, nucleus accumbens

## Abstract

The mesolimbic dopamine system is a set of subcortical brain circuits that plays a key role in reward processing, reinforcement, associative learning, and behavioral responses to salient environmental events. In our previous studies of the dopaminergic response to salient visual stimuli, we observed that dopamine release in the lateral nucleus accumbens (LNAc) of mice encoded information about the rate and magnitude of rapid environmental luminance changes from darkness. Light-evoked dopamine responses were rate-dependent, robust to the time of testing or stimulus novelty, and required phototransduction by rod and cone opsins. However, it is unknown if these dopaminergic responses also involve non-visual opsins, such as melanopsin, the primary photopigment expressed by intrinsically photosensitive retinal ganglion cells (ipRGCs). In the current study, we evaluated the role of melanopsin in the dopaminergic response to light in the LNAc using the genetically encoded dopamine sensor dLight1 and fiber photometry. By measuring light-evoked dopamine responses across a broad irradiance and wavelength range in constitutive melanopsin (*Opn4*) knockout mice, we were able to provide new insights into the ability of non-visual opsins to regulate the mesolimbic dopamine response to visual stimuli.

## Introduction

The ability to consciously perceive visual stimuli in the environment, such as an evening sunset or shadows cast by a storm cloud, is mediated by a diverse set of retinal and brain circuits. Initially, phototransduction occurs in the retina, where rod and cone photoreceptors convert incident photons into changes in neurotransmission (Kawamura and Tachibanaki, 2008). Visual information is subsequently transmitted through the retinal synaptic network (Gollisch and Meister, 2010) to retinal ganglion cells (RGCs), whose axons project to the brain via the optic nerve (Dhande and Huberman, 2014). In image forming visual pathways, RGC axons synapse in the thalamic lateral geniculate nucleus (LGN) that, in turn, innervates the primary visual cortex for higher order visual processing (Callaway, 2005). RGCs are a heterogenous neuronal population, and some are involved in other physiological responses to light outside of image formation (Dhande and Huberman, 2014; Goetz et al., 2022). Functions regulated by non-image forming visual pathways include circadian entrainment, pupillary light reflexes, gaze orientation, and light-dependent changes in mood (Beier et al., 2022; Mahoney and Schmidt, 2024). Intrinsically photosensitive retinal ganglion cells (ipRGCs) play an important role in non-image forming visual circuits (Mure, 2021) and make up approximately 1% of the total population of RGCs (Hattar et al., 2002; Hannibal et al., 2017). These neurons are able to respond to photic signals without synaptic input from rods and cones (Berson et al., 2002) because they express the photopigment melanopsin, which is encoded by the *OPN4* gene in humans and *Opn4* in the mouse (Schroeder et al., 2018; Aranda and Schmidt, 2021). Melanopsin is a light-sensitive G-protein coupled receptor (peak absorbance: 480 nm) that couples to G_aq/11_ (Newman et al., 2003). When activated by light, melanopsin initiates a phosphoinositide cascade (Graham et al., 2008) that causes calcium influx and, subsequently, changes in ipRGC firing (Do and Yau, 2013).

In rodents, ipRGCs project widely throughout the brain, and synaptic targets include the LGN, superior colliculus (SC), suprachiasmatic nucleus (SCN), perihabenular region (PHb), intergeniculate leaf (IGL), preoptic area (POA), medial amygdala, etc. (Berson et al., 2002; Güler et al., 2008; Ecker et al., 2010; Chen et al., 2011; Fernandez et al., 2018; Zhang et al., 2021). Functional studies indicate that ipRGC projections coordinate different physiological and homeostatic processes depending on their target. For example, melanopsin knockout or ablation of ipRGCs that project to the SCN perturbs entrainment of the circadian clock to changes in the day-night cycle (Freedman et al., 1999; Panda et al., 2003; Güler et al., 2008). Loss of ipRGCs projecting to the olivary pretectal nucleus (OPN) attenuates the pupillary eye reflex (Gamlin et al., 2007; Güler et al., 2008) that controls light-dependent changes in pupil size and represents a protective mechanism for the retina (Belliveau et al., 2024). ipRGCs aid in pattern recognition (Ecker et al., 2010) and modulate luxotonic (irradiance-dependent) firing in the dorsal LGN (Storchi et al., 2015). Finally, mood regulation by light, which may be dysregulated in shift workers (Scott et al., 1997) or in seasonal affective disorder (Melrose, 2015), has been attributed to an ipRGC circuit involving the PHb (Fernandez et al., 2018; Weil et al., 2022) and its downstream targets, such as the zona incerta, thalamic reticular nucleus, and the nucleus accumbens (NAc) (Fernandez et al., 2018; Milosavljevic et al., 2016; Weil et al., 2022). Of these, light-dependent changes in neurotransmission in the NAc are of particular interest, as this site is a critical node in the mesolimbic dopamine system (Carlezon and Thomas, 2009) and is a target for deep brain stimulation in clinical trials involving mood disorders (Bewernick et al., 2010, 2012).

The mesolimbic dopamine system is an evolutionarily old set of circuits that regulates motivation, appetitive behavior, and attention (Wise, 2004; Watabe-Uchida et al., 2017; Berke, 2018). In this pathway, ascending projections from the ventral midbrain respond to salient stimuli and promote motivated behavior by releasing dopamine in limbic structures, such as the NAc, amygdala, and bed nucleus of the stria terminalis (Wise, 2005; Bromberg-Martin et al., 2010; Beier et al., 2015). Dopamine is an important regulator of mood (Nestler and Carlezon, 2006) and influences sleep and arousal state via dopaminergic projections from the ventral tegmental area (VTA) to the NAc (Eban-Rothschild et al., 2016). In our previous study (Gonzalez et al., 2023), we showed that dopamine release in the lateral NAc (LNAc) encodes the rate and magnitude of dark-to-light environmental lighting transitions, such as when a lightbulb is turned on in a dark room or when an animal emerges from a darkened burrow. This ability of dopamine to signal information about rapid environmental luminance changes is wavelength-dependent at low irradiances, independent of the circadian cycle and stimulus novelty, and involves rod and cone phototransduction. It is also highly sensitive, as dopamine could be evoked by light intensities that were imperceptible to human experimenters (Gonzalez et al., 2023) but visible to nocturnal rodents with superior scotopic vision (Peirson et al., 2018). It is unknown, however, if this ability of dopamine to encode information about rapid lighting transitions involves ipRGCs.

Recently, it has been hypothesized that melanopsin-expressing ipRGCs could modulate the activity of VTA-to-NAc projections via a disynaptic circuit involving the preoptic area (Zhang et al., 2021). However, the ability of melanopsin phototransduction to influence visual stimulus-dependent striatal dopamine release has not been thoroughly tested. Previously, we began to address this topic using the genetically encoded dopamine sensor dLight1 and fiber photometry. This GFP-based sensor provides a fluorescent readout of dopamine dynamics *in vivo* in awake, behaving mice with sub-second resolution (Patriarchi et al., 2018). Using dLight1, we observed that the dopaminergic response to light was greatly attenuated, but not eliminated, in mice that lacked rod and cone G protein subunit *α*-transducin 1 and 2 (*Gnat1/2*^−/−^ mice). In contrast, melanopsin loss in *Opn4* knockout mice (*Opn4^−/−^*) did not affect the magnitude of light-evoked release dopamine (Gonzalez et al., 2023). However, a single, high intensity white light stimulus was utilized in this experiment, so little is known about the effect of melanopsin deletion on the LNAc dopaminergic response to light across a broader irradiance and wavelength range. In the current study, we addressed this knowledge gap by measuring dLight1 transients evoked by single-color LED light stimuli across the visual spectrum and a 10,000-fold irradiance range in *Opn4* knockout mice and their wildtype littermates. These experiments revealed new information about the influence of non-visual opsins on signaling within the mesolimbic dopamine system.

## Methods

### Experimental animals

Experimental subjects were adult, male and female homozygous *Opn4* knockout mice (*n* = 13) (Panda et al., 2002) and wildtype littermates (*n* = 16) that were greater than 12 weeks of age. Experiments were tested in two cohorts of 5–8 mice per genotype. Mice had *ad libitum* access to food and water, and all experimental procedures were performed during the light phase of the 14 h/10 h light/dark cycle (lights on at 0600 h, lights off at 2000 h) in the Cincinnati Children’s Hospital Medical Center (CCHMC) vivarium, as previously described (Gonzalez et al., 2023). The mice were housed in same-sex groups of two or three after fiber implantation surgeries. Following the completion of experiments, mice were transcardially perfused with 4% paraformaldehyde in phosphate-buffered saline so that the photometry fiber location could be determined histologically *post hoc*. Mice were excluded from studies if there was no photometry signal 6 weeks after surgery or if the optical fiber location was found to be outside the target area. Animal husbandry and experimental procedures involving animal subjects were approved by the Institutional Animal Care and Use Committee at CCHMC (protocol number 2023–0044) and conducted in compliance with the Guide for the Care and Use of Laboratory Animals of the National Institutes of Health.

### Surgical procedures

dLight1 surgical procedures, including viral vector injection and optical fiber implantation, were performed as previously described (Robinson et al., 2019; Gonzalez et al., 2023). *Opn4*^−/−^ and *Opn4*^+/+^ littermate control mice were anesthetized with isoflurane (1–4% in 95% oxygen/5% CO_2_) delivered through a nose cone at a rate of 1 L/min, and the skull was fixed using a stereotaxic frame (David Kopf Instruments). Chlorhexidine surgical scrub was used to clean the scalp, and the skull surface was exposed via a rostral-caudal incision in the scalp. A craniotomy hole was drilled above the location of the virus injection and photometry fiber implantation. Stereotaxic AAV injections were performed using a beveled 34 or 35-gauge microinjection needle within a 10 μL microsyringe (NanoFil, World Precision Instruments) controlled by microsyringe pump with SMARTouch Controller (UMP3T-1, World Precision Instruments). dLight1.2 was expressed in the ventral striatum via stereotaxic injection of a AAV5-hSyn-dLight1.2-WPRE vector (800 nL of virus at a titer of approximately 1 × 10^13^ viral genome/mL; Addgene catalog #111068-AAV5) in the lateral nucleus accumbens (LNAc) using the following coordinates: A/P: +1.2 mm, M/L: ±1.7 mm, D/V: −4.2 mm. Viral vectors were injected over 10 min and then allowed to diffuse throughout the tissue for 10 min before removing the injection needle slowly over several minutes. After AAV injections, a 6 mm long, 400 μm outer diameter photometry fiber with a metal ferrule (MFC_400/430–0.66_6 mm_MF1.25_FLT; Doric Lenses, Inc.) was lowered to the same coordinates and affixed to the skull with dental cement. Mice were allowed to recover on a heating pad during the post-operative period and monitored closely for 72 h following surgery. 5 mg/kg of carprofen (s.c.) was provided acutely for pain relief and once daily for 72 h after surgery.

### Fiber photometry

Fluorescent signals were monitored using an RZ10x fiber photometry system from Tucker-Davis Technologies, as previously described (Gonzalez et al., 2023). It contained a 465-nm LED for sensor excitation and a 405-nm LED for isosbestic excitation. Light was filtered and collimated using a six channel fluorescent MiniCube [FMC6_IE(400–410)_E1(460–490)_F1(500–540)_E2(555–570)_F2(580–680)_S] from Doric Lenses, Inc., which was coupled to the implanted photometry fiber via a low autofluorescence fiber optic patch cable (MFP_400/430/1100–0.57_1_FCM-MF1.25LAF; Doric Lenses, Inc.). The emission signal from 405 nm isosbestic excitation was used as a reference signal to account for motion artifacts and photo-bleaching. A first order polynomial fit was applied to align the 465 nm signal to the 405 nm signal. During experiments, the ΔF time-series trace was z-scored within epochs to account for data variability across animals and sessions, as described by Barker et al. (2017) and performed in our previous study (Gonzalez et al., 2023). dLight1 signals were aligned to stimulus onset via delivery of TTL pulses to the photometry system during light exposure experiments. Peak data (magnitude, full width at half maximum amplitude, and time to peak) was analyzed using Python.

### Visual stimulus exposure

LED light exposures were delivered to the mice from darkness in a modular conditioning chamber (Model 80015NS, Lafayette Instruments Company) placed within a light and sound attenuating enclosure that was modified to reduce the ambient light to undetectable levels. The onset of light stimuli was controlled by ABET II software (Lafayette Instrument Company), as previously described (Gonzalez et al., 2023). A TTL breakout adapter (Model 81,510) was used to synchronize stimulus delivery with the photometry recording. Individual wavelength light stimuli were generated with a Lumencor Aura III LED light engine, which was triggered via TTL inputs from the Lafayette Instruments TTL breakout adapter and controlled by ABET II. LED light power (measured at mouse level with a Thor Labs PM100D optical power meter with S130VC photodiode sensor) was modulated using the onboard Lumencor graphical user interface and, when necessary, attenuated via the use of glass neutral density filters (0.1–3.0 OD, HOYA Filter USA and/or Edmund Optics TECHSPEC filters). During each testing session, mice were dark adapted for 10 min prior to the start of testing. Ten-second single wavelength stimuli were delivered in random order with a randomized ITI between 140 and 200 s to achieve five total exposures per color per mouse for a total of 20 total exposures. Because the relative irradiance of the light stimuli could not be changed during the testing session, dopamine responses to different light intensities were recorded on different testing days.

### Statistical analysis

Statistical analysis was performed using GraphPad Prism 9 (GraphPad Software, Inc.). All statistical tests performed on data presented in the manuscript are stated in the text and/or associated figure captions. No outliers were removed during statistical analysis. Parametric tests were used unless the data set was non-normally distributed, which was determined via the D’Agostino-Pearson test for normality for two-sample comparisons and the analysis of quantile-quantile (QQ) plots when two-way repeated measures analysis of variance (ANOVA) was performed. The Shapiro–Wilk test of normality was employed in Figure 5 because the sample size was too small to perform the D’Agostino-Pearson test. When t-tests were performed, the Welch’s correction was used if sample variances were significantly different between wildtype and knockout groups (e.g., Figure 2L). When *post hoc* testing was performed after ANOVA, the Bonferroni correction was used to correct for multiple comparisons if any factor had three or more levels; otherwise, the Fisher’s Least Significant Difference (LSD) test was employed. Source data, the results of normality tests, and all statistical testing results are available in the Supplementary material. Results are presented as mean ± standard error of the mean (SEM) throughout the manuscript.

## Results

**Figure 1:**
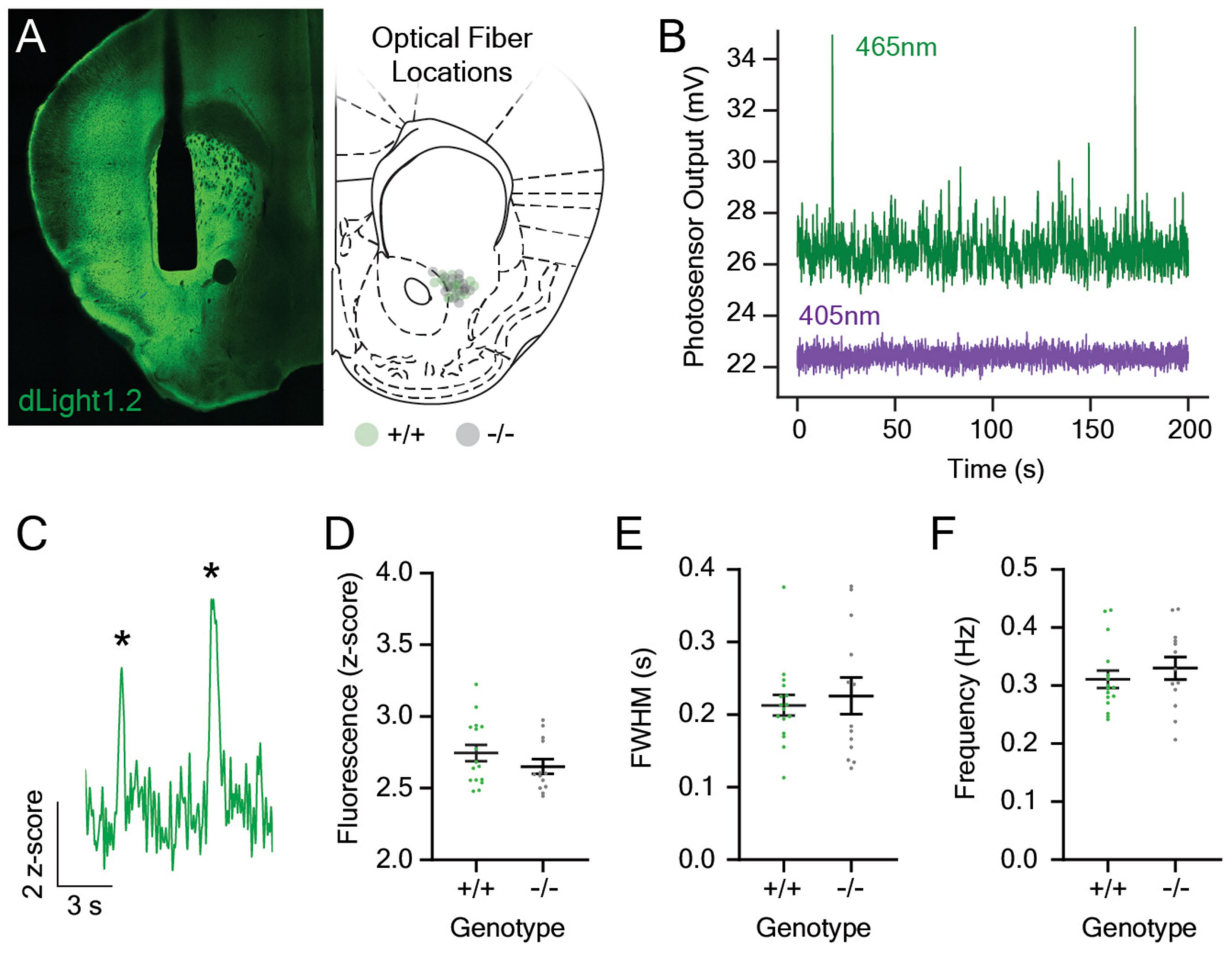
Spontaneous dopaminergic neurotransmission in the lateral nucleus accumbens in *Opn4* knockout and wildtype control mice. **(A)** (*Left*) A representative confocal image showing expression of the dLight1 sensor (*green*) and the 400 μm diameter optical fiber track in the lateral nucleus accumbens (LNAc). (*Right*) Approximate optical fiber tip locations for *Opn4*^+/+^ (*green circles*) and *Opn4*^−/−^ mice (*gray circles*) determined by *post hoc* histological analysis. **(B)** Representative fluorescence traces showing the raw dopamine-dependent dLight1 emission signal (465 nm excitation, *green*) and the isosbestic control signal (405 nm, *purple*) in the LNAc of a freely moving mouse in a dark behavioral testing chamber. **(C)** Representative fluorescence trace showing spontaneous dLight1 transients (identified by asterisks) in the LNAc. **(D–F)** There was no difference in the **(D)** magnitude (*n*_+/+_ = 16, *n*_−/−_ = 13; D’Agostino-Pearson normality test, *p*_+/+_ = 0.44, *p*_−/−_ = 0.32; unpaired t-test, *t*_27_ = 1.20, *p* = 0.24), **(E)** full width at half maximum (FWHM; *n*_+/+_ = 16, *n*_−/−_ = 13; D’Agostino-Pearson normality test, *p*_+/+_ = 0.003, *p*_−/−_ = 0.33; Mann–Whitney U test; *U* = 101, *p* = 0.91), or **(F)** frequency (*n*_+/+_ = 16, *n*_−/−_ = 13; D’Agostino-Pearson normality test, *p*_+/+_ = 0.15, *p*_−/−_ = 0.81; unpaired t-test, *t*_27_ = 0.79, *p* = 0.44) of spontaneous dLight1 transients in the LNAc in *Opn4*^−/−^ and *Opn4*^+/+^ mice.

To explore the role of melanopsin in the mesolimbic dopamine response to light stimuli, we measured stimulus-evoked changes in dLight1 fluorescence with fiber photometry in *Opn4*^−/−^ mice (*n* = 13) and their wildtype (*Opn4*^+/+^) littermates (*n* = 16). To facilitate these experiments, we performed stereotaxic surgeries to unilaterally inject an adeno-associated viral vector into the LNAc to express dLight1 in neurons (AAV5-hSyn-dLight1.2). After injection, we implanted a 400 μm diameter optical fiber in the same location so that fluorescent dopamine signals could be recorded with fiber photometry (Figure 1A, *Left*). *Post hoc* histological analysis confirmed successful targeting of the lateral NAc, and the distribution of optical fiber tip locations ranged from the lateral aspect of the NAc core to the medial NAc lateral shell region (Figure 1A, *Right*). Our fiber photometry system contained a 465 nm light emitting diode (LED) for dLight1 sensor excitation and a 405 nm LED for isosbestic illumination (Figure 1B). The dopamine-independent output of isosbestic excitation served as a reference for normalization to minimize the effects of animal motion and photobleaching on the dLight1 signal (Patriarchi et al., 2019). Following recovery from surgery and time for sensor expression, we measured spontaneous dopamine release events (Figure 1C) while mice sat in darkness in a behavioral testing chamber placed within a light and sound attenuating enclosure. No differences in the magnitude (Figure 1D), full width at half maximum amplitude (FWHM; Figure 1E), or rate (Figure 1F) of spontaneous dLight1 transients were observed between genotypes, which suggests that melanopsin loss does not affect basal dopaminergic transmission in the LNAc in the absence of a light stimulus.

**Figure 2:**
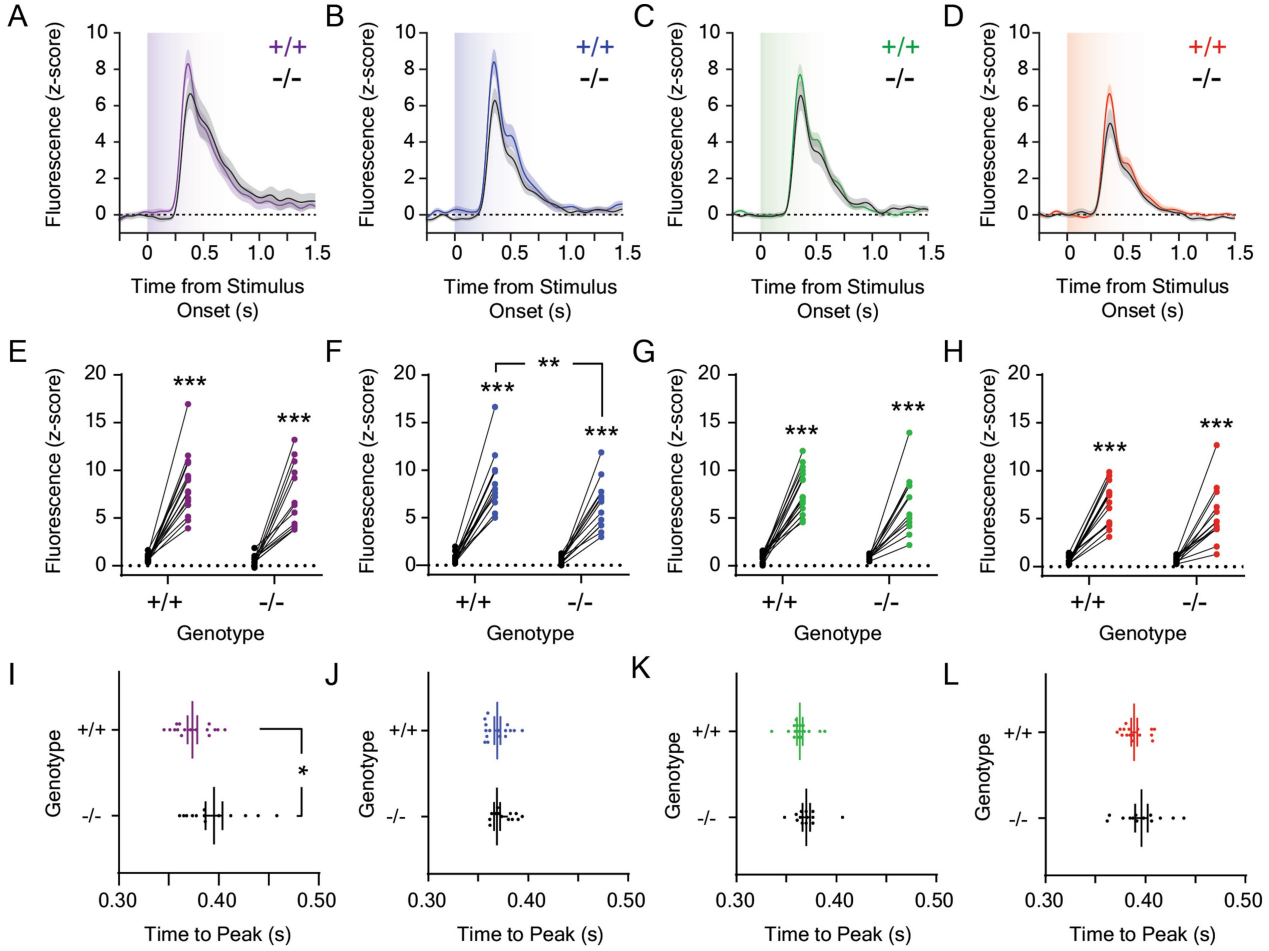
Dopamine responses to 1.0 μW/cm^2^ light in the lateral nucleus accumbens in *Opn4* knockout and wildtype control mice. Average LNAc dLight1 fluorescence traces at the onset of a ten-second **(A)** UV, **(B)** blue, **(C)** green, or **(D)** red light stimulus from darkness *±* standard error of the mean. *Opn4*^−/−^ fluorescence traces are shown in black, while *Opn4*^+/+^ traces are shown in the color of the LED stimulus. **(E–H)** Peak dLight1 responses to 1.0 μW/cm^2^, ten-second UV, blue, green, or red light stimuli relative to a pre-stimulus baseline (*black circles*) in *Opn4*^+/+^ (*left*, *‘+/+’*) and *Opn4*^−/−^ (*right, ‘−/−’*) mice. **(E)** Peak dLight1 responses were dependent on the UV light stimulus but not genotype (*n*_+/+_ = 16, *n*_−/−_ = 13; two-way repeated measures ANOVA with Fisher’s LSD *post hoc* tests; *F*_1,27_ = 1.84, *p*_genotype_ = 0.19; *F*_1,27_ = 127.9, *p*_stimulus_ < 0.001; *F*_1,27_ = 1.002, *p*_genotype x stimulus_ = 0.33). *Post hoc* tests indicated that dLight1 responses to UV light stimuli were significantly greater than baseline. **(F)** Peak dLight1 responses were dependent on the blue light stimulus and genotype (*n*_+/+_ = 16, *n*_−/−_ = 13; two RM ANOVA with Fisher’s LSD *post hoc* tests; *F*_1,27_ = 4.65, *p*_genotype_ = 0.040; *F*_1,27_ = 198.3, *p*_stimulus_ < 0.001; *F*_1,27_ = 4.55, *p*_genotype x stimulus_ = 0.042). *Post hoc* tests indicated that dLight1 responses to blue light stimuli were significantly greater than baseline and smaller in *Opn4*^−/−^ mice relative to *Opn4*^+/+^ controls. **(G)** Peak dLight1 responses were dependent on the green light stimulus but not genotype (*n*_+/+_ = 16, *n*_−/−_ = 13; two-way repeated measures ANOVA with Fisher’s LSD *post hoc* tests; *F*_1,27_ = 1.44, *p*_genotype_ = 0.24; *F*_1,27_ = 155.2, *p*_stimulus_ < 0.001; *F*_1,27_ = 1.04, *p*_genotype x stimulus_ = 0.32). *Post hoc* tests indicated that dLight1 responses to green light stimuli were significantly greater than baseline. **(H)** Peak dLight1 responses were dependent on the red light stimulus but not genotype (*n*_+/+_ = 16, *n*_−/−_ = 13; two-way repeated measures ANOVA with Fisher’s LSD *post hoc* tests; *F*_1,27_ = 1.94, *p*_genotype_ = 0.18; *F*_1,27_ = 131.5, *p*_stimulus_ < 0.001; *F*_1,27_ = 2.87, *p*_genotype x stimulus_ = 0.10). *Post hoc* tests indicated that dLight1 responses to red light stimuli were significantly greater than baseline. **(I–L)** Analysis of the time to peak dopamine release from the onset of the 1.0 mW/cm^2^, ten-second UV, blue, green, or red light stimulus. **(I)** The time to the dLight1 peak evoked by UV light was significantly longer in *Opn4^−/−^* mice relative to *Opn4^+/+^* controls (*n*_+/+_ = 16, *n*_−/−_ = 13; D’Agostino-Pearson normality test, *p*_+/+_ = 0.22, *p*_−/−_ = 0.32; unpaired t-test, *t*_27_ = 2.34, *p* = 0.027). **(J)** The time to the dLight1 peak evoked by blue light was not significantly different between *Opn4^−/−^* and *Opn4^+/+^* mice (*n*_+/+_ = 16, *n*_−/−_ = 13; D’Agostino-Pearson normality test, *p*_+/+_ = 0.36, *p*_−/−_ = 0.36; unpaired t-test, *t*_27_ = 1.70, *p* = 0.10). **(K)** The time to the dLight1 peak evoked by green light was not significantly different between *Opn4^−/−^* and *Opn4^+/+^* mice (*n*_+/+_ = 16, *n*_−/−_ = 13; D’Agostino-Pearson normality test, *p*_+/+_ = 0.26, *p*_−/−_ = 0.005; Mann–Whitney U test, *U* = 67, *p* = 0.11). **(L)** The time to the dLight1 peak evoked by red light was not significantly different between *Opn4^−/−^* and *Opn4^+/+^* mice (*n*_+/+_ = 16, *n*_−/−_ = 13; D’Agostino-Pearson normality test, *p*_+/+_ = 0.31, *p*_−/−_ = 0.87; unpaired t-test with Welch’s correction, *t*_17.7_ = 1.03, *p* = 0.32). In all panels, * indicates *p* < 0.05, ** indicates *p* < 0.01, and *** indicates *p* < 0.001.

**Figure 3:**
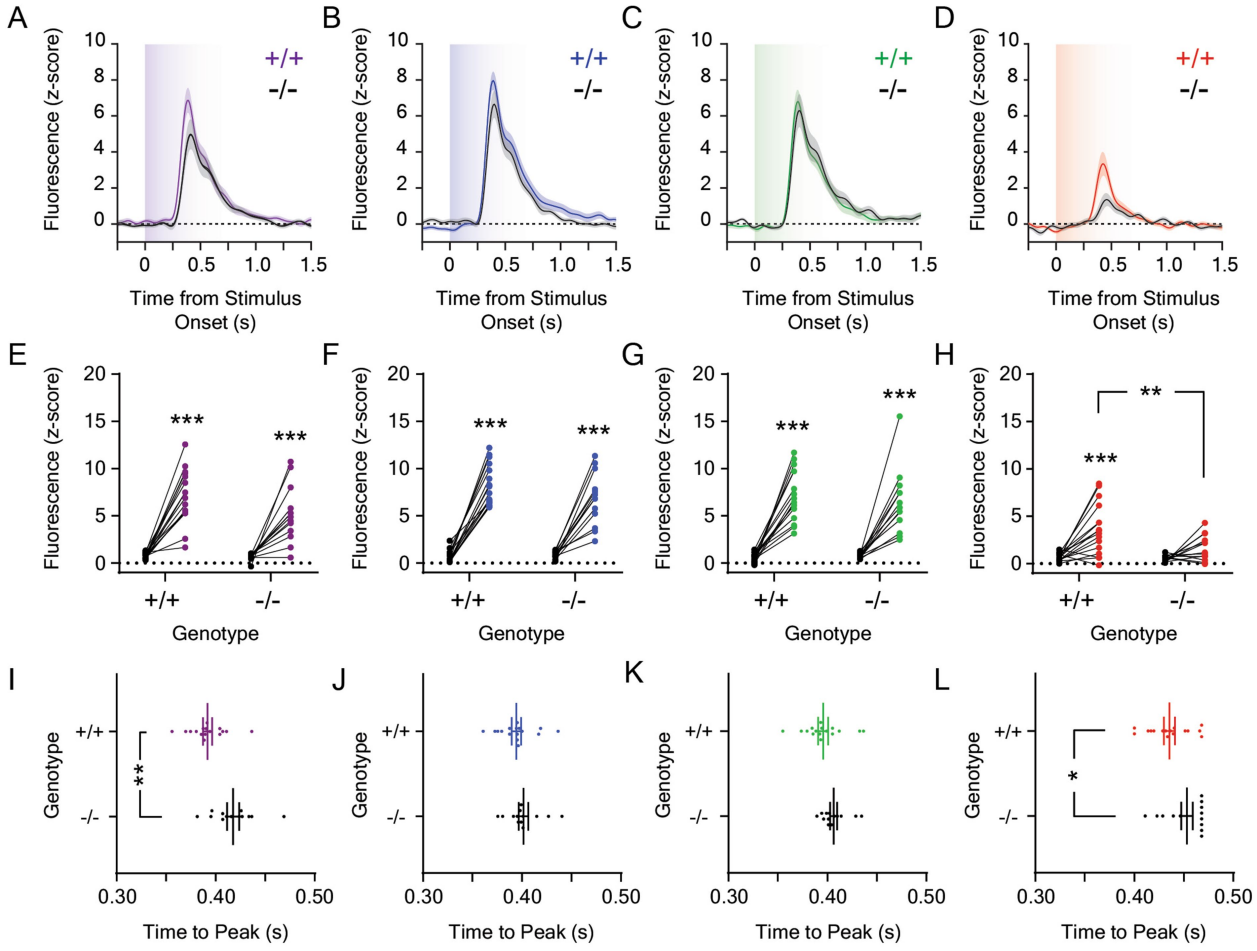
Dopamine responses to 0.001 μW/cm^2^ light in the lateral nucleus accumbens in *Opn4* knockout and wildtype control mice. Average LNAc dLight1 fluorescence traces at the onset of a ten-second **(A)** UV, **(B)** blue, **(C)** green, or **(D)** red light stimulus from darkness *±* standard error of the mean. *Opn4*^−/−^ fluorescence traces are shown in black, while *Opn4*^+/+^ traces are shown in the color of the LED stimulus. **(E–H)** Peak dLight1 responses to 0.001 μW/cm^2^, ten-second UV, blue, green, or red light stimuli relative to a pre-stimulus baseline (*black circles*) in *Opn4*^+/+^ (*left*, *‘+/+’*) and *Opn4*^−/−^ (*right, ‘−/−’*) mice. **(E)** Peak dLight1 responses were dependent on the UV light stimulus but not genotype (*n*_+/+_ = 16, *n*_−/−_ = 13; two-way repeated measures ANOVA with Fisher’s LSD *post hoc* tests; *F*_1,27_ = 3.58, *p*_genotype_ = 0.069; *F*_1,27_ = 97.3, *p*_stimulus_ < 0.001; *F*_1,27_ = 2.63, *p*_genotype x stimulus_ = 0.12). *Post hoc* tests indicated that dLight1 responses to UV light stimuli were significantly greater than baseline. **(F)** Peak dLight1 responses were dependent on the blue light stimulus but not genotype (*n*_+/+_ = 16, *n*_−/−_ = 13; two-way repeated measures ANOVA with Fisher’s LSD *post hoc* tests; *F*_1,27_ = 1.48, *p*_genotype_ = 0.23; *F*_1,27_ = 216.5, *p*_stimulus_ < 0.001; *F*_1,27_ = 3.59, *p*_genotype x stimulus_ = 0.069). *Post hoc* tests indicated that dLight1 responses to blue light stimuli were significantly greater than baseline. **(G)** Peak dLight1 responses were dependent on the green light stimulus but not genotype (*n*_+/+_ = 16, *n*_−/−_ = 13; two-way repeated measures ANOVA with Fisher’s LSD *post hoc* tests; *F*_1,27_ = 0.38, *p*_genotype_ = 0.54; *F*_1,27_ = 110.6, *p*_stimulus_ < 0.001; *F*_1,27_ = 0.46, *p*_genotype x stimulus_ = 0.50). *Post hoc* tests indicated that dLight1 responses to green light stimuli were significantly greater than baseline. **(H)** Peak dLight1 responses were dependent on the red light stimulus and genotype (*n*_+/+_ = 16, *n*_−/−_ = 13; two-way repeated measures ANOVA with Fisher’s LSD *post hoc* tests; *F*_1,27_ = 6.56, *p*_genotype_ = 0.016; *F*_1,27_ = 22.9, *p*_stimulus_ < 0.001; *F*_1,27_ = 4.40, *p*_genotype x stimulus_ = 0.046). *Post hoc* tests indicated that the dLight1 response to red light in *Opn4*^+/+^ mice was significantly greater than baseline and *Opn4*^−/−^ littermates. No significant red light-evoked dLight1 transient was observed in *Opn4*^−/−^ mice relative to baseline. **(I–L)** Analysis of the time to peak dopamine release from the onset of the 0.001 μW/cm^2^, ten-second UV, blue, green, or red light stimulus. **(I)** The time to the dLight1 peak evoked by UV light was significantly longer in *Opn4^−/−^* mice relative to *Opn4^+/+^* controls (*n*_+/+_ = 16, *n*_−/−_ = 13; D’Agostino-Pearson normality test, *p*_+/+_ = 0.28, *p*_−/−_ = 0.27; unpaired t-test, *t*_27_ = 3.39, *p* = 0.002). **(J)** The time to the dLight1 peak evoked by blue light was not significantly different between *Opn4^−/−^* and *Opn4^+/+^* mice (*n*_+/+_ = 16, *n*_−/−_ = 13; D’Agostino-Pearson normality test, *p*_+/+_ = 0.42, *p*_−/−_ = 0.18; unpaired t-test, *t*_27_ = 1.07, *p* = 0.30). **(K)** The time to the dLight1 peak evoked by green light was not significantly different between *Opn4^−/−^* and *Opn4^+/+^* mice (*n*_+/+_ = 16, *n*_−/−_ = 13; D’Agostino-Pearson normality test, *p*_+/+_ = 0.51, *p*_−/−_ = 0.11; unpaired t-test, *t*_27_ = 1.64, *p* = 0.11). **(L)** The time to the dLight1 peak evoked by red light was longer in *Opn4^−/−^* mice relative to *Opn4^+/+^* controls (*n*_+/+_ = 16, *n*_−/−_ = 13; D’Agostino-Pearson normality test, *p*_+/+_ = 0.71, *p*_−/−_ = 0.24; unpaired t-test, *t*_27_ = 2.19, *p* = 0.037). In all panels, * indicates *p* < 0.05, ** indicates *p* < 0.01, and *** indicates *p* < 0.001.

We next measured light-evoked dopamine release in the LNAc in *Opn4*^−/−^ and *Opn4*^+/+^ mice. dLight1 signals were recorded during ten-second light exposures from darkness delivered via a TTL-triggerable light engine that contained band pass-filtered ultraviolet (UV; 360 nm/28 nm), blue (475 nm/28 nm), green (555 nm/28 nm), and red (635 nm/22 nm) LEDs (see Supplementary Figure S1 for spectrometer characterization of each LED output). For each stimulus exposure, we calculated the magnitude and time to peak stimulus-evoked dopamine release, which are both irradiance-dependent response features in the mouse LNAc. Specifically, increasing the intensity of the light stimulus non-linearly increased the magnitude of the dopamine transient and decreased the response latency in previous studies (Gonzalez et al., 2023). While the ethological function of this luxotonic dopamine release is not fully understood, it does not relate to mouse movement, as we have not observed statistically significant LNAc dopamine release at the onset of locomotion or during the performance of vigorous motor actions, such as escape from visual threats (Fisher et al., 2025). In each experiment, we compared light-evoked dLight1 peaks to a pre-stimulus baseline defined as the largest dLight1 peak that occurred during a one-second period before stimulus delivery, which approximated the amplitude of spontaneous neurotransmission and/or fluorescent noise. We also calculated the FWHM of the evoked dopamine transient to describe its duration. Mice were exposed to 1 μW/cm^2^ (Figure 2) or 0.001 μW/cm^2^ (Figure 3) stimuli from darkness; these irradiances were chosen based on the irradiance-response curve previously established for white light-evoked LNAc dopamine release in mice (Gonzalez et al., 2023). While 1 μW/cm^2^ is a saturating stimulus, 0.001 μW/cm^2^ light evokes approximately 50% of the maximum LNAc dopamine response.

When mice were exposed to ten-second, 1 μW/cm^2^, UV (Figure 2A), blue (Figure 2B), green (Figure 2C), or red (Figure 2D) light stimuli from darkness, large phasic dopamine transients were observed exclusively at the onset of each dark-to-light transition that were statistically significant relative to baseline (Figures 2E–H). When dopamine transients were compared between genotypes, a significant reduction in the peak dLight1 response to blue light was observed in *Opn4*^−/−^ mice relative to *Opn4*^+/+^ littermates (Figure 2F). When all wavelengths were compared, peak dopamine responses were dependent on wavelength but not genotype (Supplementary Figure S2A). Independent of genotype, the dopamine response to 1 μW/cm^2^ red light was significantly smaller than the response to other colors. We also observed that the time to the dLight1 peak (Figures 2I–L) evoked by UV light was significantly greater in *Opn4*^−/−^ mice relative to controls (Figure 2I), and across all wavelengths, the effect of wavelength on peak time was genotype-dependent (Supplementary Figure S2B). Within *Opn4*^+/+^ mice, the time to the dLight1 peak evoked by blue and green stimuli was shorter than the time to dLight1 peaks evoked by red and UV stimuli, which, in turn, were different from each other (Supplementary Figure S2B, *magenta symbols*). Within *Opn4*^−/−^ mice, the time to the dLight1 peak evoked by blue and green stimuli was shorter than the time to dLight1 peaks evoked by red and UV stimuli, which were not different from each other (Supplementary Figure S2B, *black symbols*). No differences in the light-evoked dLight1 transient FWHM were observed between *Opn4*^−/−^ and *Opn4*^+/+^ mice, and this dopamine transient characteristic was wavelength-dependent when analyzed across stimuli (Supplementary Figure S2C). Independent of genotype, the FWHM of dopamine transients evoked by UV light was significantly greater than those evoked by blue and red light.

Next, we analyzed the effects of melanopsin knockout on the LNAc dopamine response to 0.001 μW/cm^2^ light stimuli (Figures 3A–D), which evoked phasic dopamine release at stimulus onset that was greater than baseline for UV (Figure 3E), blue (Figure 3F), and green (Figure 3G) light stimuli. While the dopamine response to red light was greater than baseline in *Opn4*^+/+^ mice, it did not reach statistical significance in *Opn4*^−/−^ mice (Figure 3H). Likewise, the peak dopaminergic response to red light, but not other wavelengths, was decreased in *Opn4*^−/−^ mice relative to littermate controls (Figure 3H). When all wavelengths were compared, the peak dopamine response to 0.001 μW/cm^2^ light was dependent on wavelength but not genotype (Supplementary Figure S3A). Independent of genotype, the dopaminergic response to red light was smaller than the response to other colors, and the dLight1 transient evoked by the UV light stimulus was smaller than the response to blue but not green stimuli. When we analyzed the time to the dLight1 peak for individual colors (Figures 3I–L), we observed that peaks evoked by UV (Figure 3I) and red light (Figure 3L) occurred significantly later in *Opn4*^−/−^ mice relative to littermate controls. When all wavelengths were compared, there were significant main effects of wavelength (*p* < 0.001) and genotype (*p* = 0.01) on time to peak that did not interact (Supplementary Figure S3B). Independent of genotype, the dopaminergic transient evoked by red light had a longer time to peak than the response to other colors. No differences in the FWHM of dLight1 transients evoked by 0.001 μW/cm^2^ light stimuli were observed between genotypes, and these responses were-wavelength dependent (Supplementary Figure S3C). Independent of genotype, the FWHM of dopamine transients evoked red light was significantly smaller than those evoked by UV, blue, and green light.

**Figure 4:**
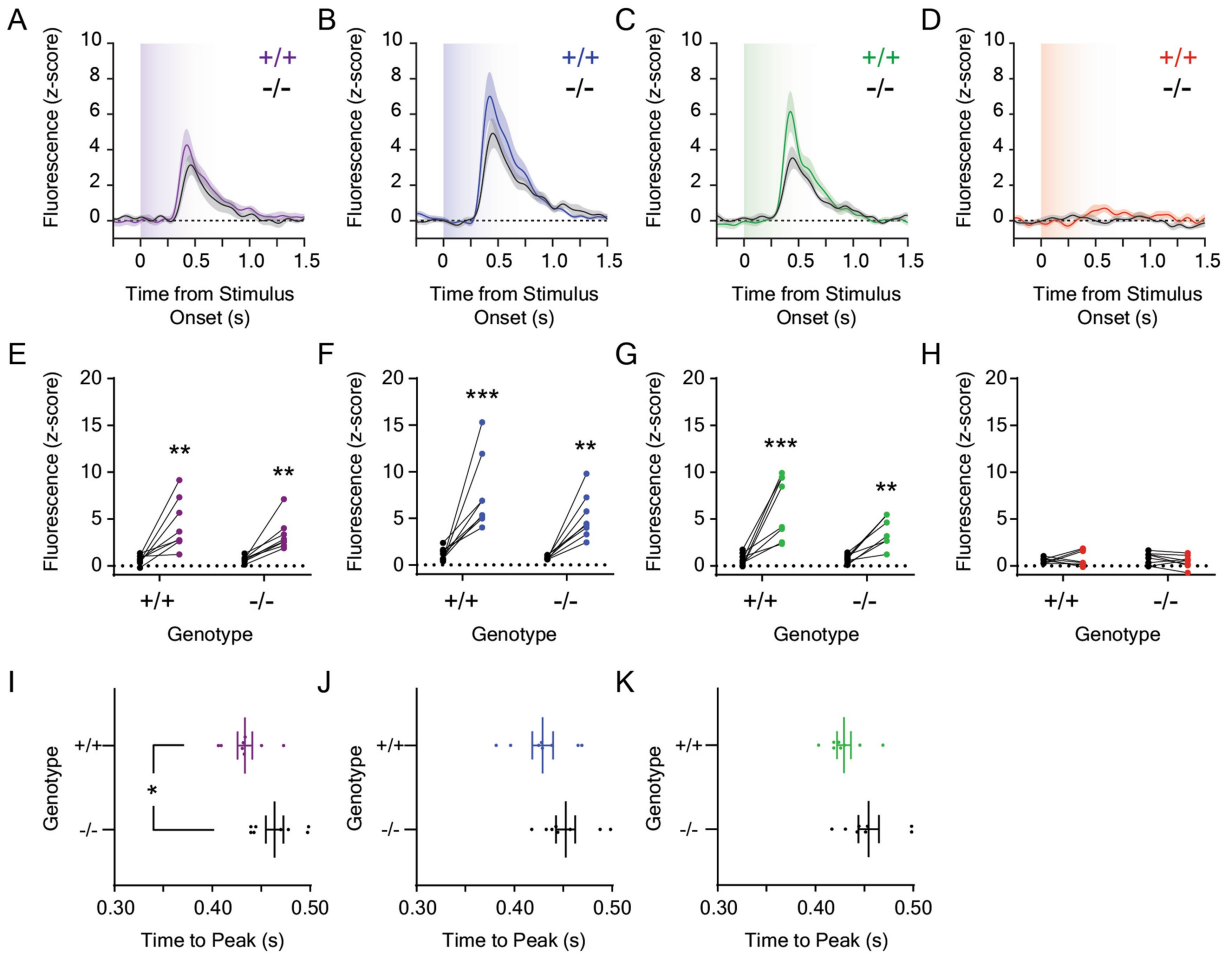
Dopamine responses to 0.0001 μW/cm^2^ light in the lateral nucleus accumbens in *Opn4* knockout and wildtype control mice. Average LNAc dLight1 fluorescence traces at the onset of a ten-second **(A)** UV, **(B)** blue, **(C)** green, or **(D)** red light stimulus from darkness *±* standard error of the mean. *Opn4*^−/−^ fluorescence traces are shown in black, while *Opn4*^+/+^ traces are shown in the color of the LED stimulus. **(E–H)** Peak dLight1 responses to 0.0001 μW/cm^2^, ten-second UV, blue, green, or red light stimuli relative to a pre-stimulus baseline (*black circles*) in *Opn4*^+/+^ (*left*, *‘+/+’*) and *Opn4*^−/−^ (*right, ‘−/−’*) mice. **(E)** Peak dLight1 responses were dependent on the UV light stimulus but not genotype (*n*_+/+_ = 8, *n*_−/−_ = 8; two-way repeated measures ANOVA with Fisher’s LSD *post hoc* tests; *F*_1,14_ = 0.87, *p*_genotype_ = 0.37; *F*_1,14_ = 31.7, *p*_stimulus_ < 0.001; *F*_1,14_ = 0.92, *p*_genotype x stimulus_ = 0.35). *Post hoc* tests indicated that dLight1 responses to UV light stimuli were significantly greater than baseline. **(F)** Peak dLight1 responses were dependent on the blue light stimulus but not genotype (*n*_+/+_ = 8, *n*_−/−_ = 8; two-way repeated measures ANOVA with Fisher’s LSD *post hoc* tests; *F*_1,14_ = 3.29, *p*_genotype_ = 0.091; *F*_1,14_ = 37.8, *p*_stimulus_ < 0.001; *F*_1,14_ = 1.35, *p*_genotype x stimulus_ = 0.27). *Post hoc* tests indicated that dLight1 responses to blue light stimuli were significantly greater than baseline. **(G)** Peak dLight1 responses were dependent on the green light stimulus but not genotype (*n*_+/+_ = 8, *n*_−/−_ = 8; two-way repeated measures ANOVA with Fisher’s LSD *post hoc* tests; *F*_1,14_ = 2.99, *p*_genotype_ = 0.11; *F*_1,14_ = 42.9, *p*_stimulus_ < 0.001; *F*_1,14_ = 3.65, *p*_genotype x stimulus_ = 0.077). *Post hoc* tests indicated that dLight1 responses to green light stimuli were significantly greater than baseline. **(H)** Peak dLight1 responses were not dependent on the red light stimulus or genotype (*n*_+/+_ = 8, *n*_−/−_ = 8; two-way repeated measures ANOVA with Fisher’s LSD *post hoc* tests; *F*_1,14_ = 0.008, *p*_genotype_ = 0.93; *F*_1,14_ = 0.12, *p*_stimulus_ = 0.73; *F*_1,14_ = 1.56, *p*_genotype x stimulus_ = 0.23). **(I–K)** Analysis of the time to peak dopamine release from the onset of the 0.0001 μW/cm^2^, ten-second UV, blue, or green light stimulus. No time to peak or FWHM data is shown for red light stimuli given that significant light-evoked dLight1 peaks were not detected for this color. **(I)** The time to the dLight1 peak evoked by UV light was significantly longer in *Opn4^−/−^* mice compared to *Opn4^+/+^* littermates (*n*_+/+_ = 8, *n*_−/−_ = 8; D’Agostino-Pearson normality test, *p*_+/+_ = 0.57, *p*_−/−_ = 0.27; unpaired t-test, *t*_14_ = 2.57, *p* = 0.022). **(J)** The time to the dLight1 peak evoked by blue light was not significantly different between *Opn4^−/−^* and *Opn4^+/+^* mice (*n*_+/+_ = 8, *n*_−/−_ = 8; D’Agostino-Pearson normality test, *p*_+/+_ = 0.94, *p*_−/−_ = 0.56; unpaired t-test, *t*_14_ = 1.63, *p* = 0.13). **(K)** The time to the dLight1 peak evoked by green light was not significantly different between *Opn4^−/−^* and *Opn4^+/+^* mice (*n*_+/+_ = 8, *n*_−/−_ = 8; D’Agostino-Pearson normality test, *p*_+/+_ = 0.17, *p*_−/−_ = 0.60; unpaired t-test, *t*_14_ = 2.00, *p* = 0.065). In all panels, * indicates *p* < 0.05, ** indicates *p* < 0.01, and *** indicates *p* < 0.001.

Given that the mesolimbic dopamine response to 0.001 μW/cm^2^ blue and green light remained robust despite a 1,000-fold reduction in stimulus irradiance, we reduced the stimulus intensity an additional 10-fold and measured dLight1 transients evoked by 0.0001 μW/cm^2^ light stimuli from darkness (Figure 4; *n* = 8 *Opn4*^−/−^ and 8 *Opn4*^+/+^ mice). dLight1 transients were observed at the onset of ten-second UV (Figure 4A), blue (Figure 4B), and green (Figure 4C) but not red (Figure 4D) light stimuli that were not significantly different between *Opn4*^−/−^ and *Opn4*^+/+^ mice. When all wavelengths were compared, the peak dopamine response to 0.0001 μW/cm^2^ light was dependent on wavelength but not genotype (Supplementary Figure S4A). Independent of genotype, the dopaminergic response to red light was smaller than the response to other colors, and the dLight1 transient evoked by the UV light stimulus was smaller than the response to blue stimuli. When we analyzed the time to the dLight1 peak for individual colors (Figures 4I–K), we observed that peaks evoked by UV (Figure 4I) occurred significantly later in *Opn4*^−/−^ mice relative to littermate controls. Across all wavelengths, the time to the dLight1 peak was dependent on genotype but not wavelength (Supplementary Figure S4B). No differences in the dLight1 transient FWHM evoked by 0.0001 μW/cm^2^ light stimuli were observed between genotypes, and these responses were wavelength dependent (Supplementary Figure S4C). However, no significant differences in FWHM were observed between wavelengths when *post hoc* testing was performed.

**Figure 5:**
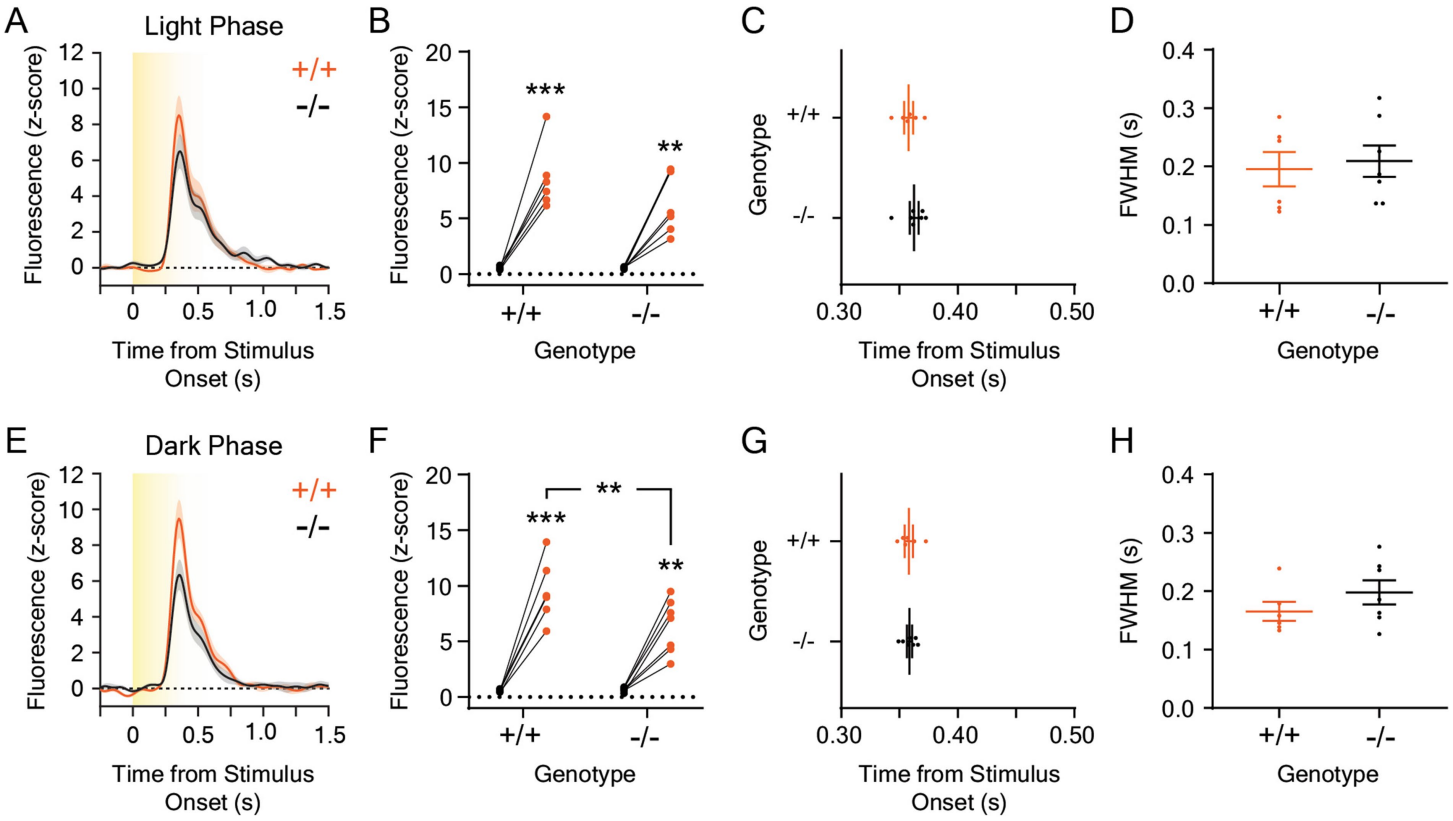
Dopamine responses to 5 μW/cm^2^ white light stimuli in the lateral nucleus accumbens in *Opn4* knockout and wildtype control mice at different times in the day-night cycle. **(A)** Average LNAc dLight1 fluorescence traces at the onset of a ten-second, 5 μW/cm^2^ white light stimulus from darkness measured 2 h after the start of the light phase (0800 h, *“Light Phase”*) of the day-night cycle ± standard error of the mean. *Opn4*^−/−^ fluorescence traces are shown in black, while *Opn4*^+/+^ traces are shown in orange. **(B)** Peak dLight1 responses were dependent on the light stimulus but not genotype when tested during the light phase (*n*_+/+_ = 6, *n*_−/−_ = 7; two-way repeated measures ANOVA with Fisher’s LSD *post hoc* tests; *F*_1,11_ = 1.60, *p*_genotype_ = 0.23; *F*_1,11_ = 80.6, *p*_stimulus_ < 0.001; *F*_1,11_ = 1.88, *p*_genotype x stimulus_ = 0.20). **(C)** The time to the dLight1 peak evoked by white light during the light phase was not significantly different between *Opn4*^−/−^ and *Opn4*^+/+^ mice (*n*_+/+_ = 6, *n*_−/−_ = 7; Shapiro–Wilk normality test, *p*_+/+_ = 0.99, *p*_−/−_ = 0.17; unpaired t-test, *t*_11_ = 0.86, *p* = 0.41). **(D)** The full width at half maximum amplitude (FWHM) evoked by the white light stimulus was not significantly different between *Opn4*^−/−^ and *Opn4*^+/+^ mice during the light phase of the day-night cycle (*n*_+/+_ = 6, *n*_−/−_ = 7; Shapiro–Wilk normality test, *p*_+/+_ = 0.12, *p*_−/−_ = 0.36; unpaired t-test, *t*_11_ = 0.35, *p* = 0.73). **(E)** Average LNAc dLight1 fluorescence traces at the onset of a ten-second, 5 μW/cm^2^ white light stimulus from darkness measured 2 h after the start of the dark phase (2200 h, *“Dark Phase”*) of the day-night cycle ± standard error of the mean. *Opn4*^−/−^ fluorescence traces are shown in black, while *Opn4*^+/+^ traces are shown in orange. **(F)** The effect of stimulus on peak dLight1 responses to light was dependent upon genotype when mice were tested during the dark phase of the day-night cycle (*n*_+/+_ = 6, *n*_−/−_ = 7; two-way repeated measures ANOVA with Fisher’s LSD *post hoc* tests; *F*_1,11_ = 4.08, *p*_genotype_ = 0.069; *F*_1,11_ = 108.3, *p*_stimulus_ < 0.001; *F*_1,11_ = 5.63, *p*_genotype x stimulus_ = 0.037). *Post hoc* tests indicated that dLight1 responses to light stimuli were significantly greater than baseline and smaller in *Opn4*^−/−^ mice relative to *Opn4*^+/+^ controls. **(G)** The time to the dLight1 peak evoked by white light during the dark phase was not significantly different between *Opn4*^−/−^ and *Opn4*^+/+^ mice (*n*_+/+_ = 6, *n*_−/−_ = 7; Shapiro–Wilk normality test, *p*_+/+_ = 0.69, *p*_−/−_ = 0.79; unpaired t-test, *t*_11_ = 0.13, *p* = 0.90). **(H)** The FWHM evoked by the white light stimulus was not significantly different between *Opn4*^−/−^ and *Opn4*^+/+^ mice during the dark phase of the day-night cycle (*n*_+/+_ = 6, *n*_−/−_ = 7; Shapiro–Wilk normality test, *p*_+/+_ = 0.11, *p*_−/−_ = 0.70; unpaired t-test, *t*_11_ = 1.21, *p* = 0.25). In all panels, * indicates *p* < 0.05, ** indicates *p* < 0.01, and *** indicates *p* < 0.001.

The experiments performed in this study were conducted approximately 2 h after the start of the light phase of the day-night cycle in our vivarium (at 0800 h). Because melanopsin plays a critical role in circadian entrainment and light-dependent changes in animal behavior (Panda et al., 2002; Güler et al., 2008; Fernandez et al., 2018), we hypothesized that the dopaminergic response to rapid, environmental luminance changes may be different at different times of the day in *Opn4*^−/−^ mice. To test this, we recorded dopamine release evoked by five, ten-second, 5.0 μW/cm^2^, white, overhead LED light stimuli from darkness at 0800 h or at 2200 h, which is 2 h after the start of dark phase in our vivarium (Figure 5; *n* = 7 *Opn4*^−/−^ and 6 *Opn4*^+/+^ mice). This white LED stimulus intensity was chosen because it was previously used to examine the LNAc dopaminergic response to light at different times of the day (Gonzalez et al., 2023). During the dark phase of the day-night cycle, light stimuli evoked large dopamine transients only at the onset of the stimulus (Figure 5A) that were significantly different from baseline (Figure 5B). However, no differences in magnitude (Figure 5B), time to peak (Figure 5C), or FWHM (Figure 5D) of the dopaminergic response to a white LED stimulus were observed between *Opn4*^−/−^ mice and their *Opn4*^+/+^ littermates at 0800 h. When mice were tested during the dark phase of the day-night cycle, we also observed high amplitude dLight1 transients that occurred exclusively at the onset of the dark-to-light transition (Figure 5E) that were significantly larger than baseline (Figure 5F). Additionally, the LNAc dopamine response to light was significantly smaller in *Opn4*^−/−^ mice relative to *Opn4*^+/+^ littermates at 2200 h. No differences in the time to peak (Figure 5G) or FWHM (Figure 5H) of the light-evoked dopamine transient was observed between genotypes. Within mice, there was no significant effect of the time of testing on the dLight1 peak magnitude (paired t-test; *Opn4*^+/+^: *t*_5_ = 1.45, *p* = 0.21; *Opn4*^−/−^: *t*_6_ = 0.36, *p* = 0.73), time to peak (paired t-test; *Opn4*^+/+^: *t*_5_ = 0.063, *p* = 0.95; *Opn4*^−/−^: *t*_6_ = 1.56, *p* = 0.17), or FWHM (paired t-test; *Opn4*^+/+^: *t*_5_ = 1.35, *p* = 0.24; *Opn4*^+/+^: *t*_6_ = 0.89, *p* = 0.41), confirming previous findings (Gonzalez et al., 2023). Therefore, the time of day may significantly affect phenotypic expression in melanopsin knockout mice, even if the LNAc dopaminergic response to light does not vary across the day-night cycle.

## Discussion

In the present study, we used the genetically encoded sensor dLight1 to further explore the role of melanopsin expressed by ipRGCs in the mesolimbic dopamine response to light. While melanopsin knockout did not significantly affect spontaneous dopaminergic neurotransmission, subtle differences in light-evoked dopamine release were observed in the LNAc of *Opn4*^−/−^ mice. Across all wavelengths and irradiances, there was no significant effect of genotype on the amplitude or half-width of the dLight1 transient that occurred at stimulus onset. However, there was a trend toward decreased dLight1 peak amplitude across experiments (~20% reduction) that reached statistical significance for some comparisons. Melanopsin loss also increased the time to peak light-evoked dopamine release at all irradiances tested, particularly for UV light. These findings indicate that melanopsin loss has a small but significant effect on the mesolimbic dopamine response to light, which could occur through several mechanisms. First, it could occur via perturbations in the ability of ipRGCs to convey photic signals to their downstream synaptic targets, such as the superior colliculus, which controls the striatal dopaminergic response to light in rodents (Redgrave et al., 2010). It could also alter signal processing in retinal microcircuits, where retrograde signaling by M1 ipRGCs affect light adaptation via connections to dopaminergic amacrine cells (Prigge et al., 2016). The observed results could have a developmental origin, as melanopsin signaling controls rod photoreceptor number by promoting apoptosis of precursor cells prior to eye opening (D’Souza et al., 2024). Future research will be required to address these hypotheses, as well as determine why the latency of the dopaminergic response to light is irradiance-dependent, a phenomenon that may relate to Bloch’s law of temporal summation in photoreceptors (Scharnowski et al., 2007; Donner, 2021) and be influenced by electrical coupling between rod and cone photoreceptors (Pasquale et al., 2020; Sladek and Thoreson, 2023).

In our previous work, knocking out *Gnat1* and *2* to eliminate *α*-transducin 1 and 2 expression and, subsequently, opsin signaling in rod and cone photoreceptors (Hargrave and McDowell, 1992) robustly attenuated the magnitude of the dopamine response to light. However, some sensitivity to UV and blue light was observed in *Gnat1/2*^−/−^ mice during dLight1 experiments, which we hypothesized could be caused by signal transduction by non-visual opsins. Given that melanopsin loss only had a modest effect on peak dLight1 response to light stimuli in the current study, it is doubtful that this opsin could account for the observed results in *Gnat1/2* double knockout mice. More likely, incomplete attenuation of 360 nm and 475 nm light-evoked dopamine release in *Gnat1/2^−/−^* mice was caused by residual rod-based photoreception, which has been reported in this transgenic model (Allen et al., 2010). A better approach to exploring the role of rods and cones in the dopaminergic encoding of rapid environmental luminance changes may involve the use of *Gnat1*^−/−^; *Cnga3*^−/−^ double knockout mice, whose only functional photopigment is melanopsin (Altimus et al., 2008). It is also important to acknowledge that while melanopsin is the primary photopigment expressed by ipRGCs, *Opn4* knockout does not necessarily abolish ipRGC activity, as these neurons receive input from rods and cones through the retinal synaptic network (Dacey et al., 2005; Wong et al., 2007; Schmidt and Kofuji, 2010), which greatly influences their ability to encode irradiance (Lall et al., 2010). Thus, determining the role of non-image forming pathways in the mesolimbic dopamine response to light would require ablation of ipRGCs using viral or transgenic approaches. This could be achieved by performing dLight1 experiments in mice that express an attenuated diphtheria toxin A subunit transgene from the endogenous *Opn4* locus, which causes near complete ipRGC loss when bred to homozygosity (Güler et al., 2008).

One limitation of the current study is that it did not identify the neural circuits that translate photoreception into changes in striatal dopamine release. Several previous studies have shown that the dopaminergic response to light in rodents involves the superior colliculus (Comoli et al., 2003; Dommett et al., 2005; Zhou et al., 2019; Solié et al., 2022; Li et al., 2024), which receives direct input from retinal ganglion cells, including ipRGCs (Cai et al., 2021; Gehr et al., 2023). The VTA – the main dopaminergic input to the nucleus accumbens – receives both glutamatergic (Geisler et al., 2007; Evans et al., 2018; Zhou et al., 2019; Li et al., 2024) and GABAergic (Zhang et al., 2019; Solié et al., 2022; Lei et al., 2024) input from the SC that can regulate wakefulness (Zhang et al., 2019), head orientation (Solié et al., 2022; Poisson et al., 2024), and the defensive response to threatening looming discs that simulate predator approach from above (Zhou et al., 2019). Optogenetic activation of ventral SC neurons or their VTA terminals causes time-locked firing of dopaminergic neurons (Zhou et al., 2019; Solié et al., 2022) or dopamine release in the LNAc (Robinson et al., 2019), as well as escape behavior in the absence of a threatening visual stimulus (Robinson et al., 2019; Zhou et al., 2019). The SC also innervates the substantia nigra pars compacta (SNc) (Dommett et al., 2005), which sends dopaminergic projections to the dorsal striatum and, to a lesser extent, the LNAc (Beier et al., 2015; Lerner et al., 2015). These tectonigral projections regulate visually directed prey capture (Huang et al., 2021) and cue-related pose adjustments (Poisson et al., 2024). Therefore, visual stimulus-evoked dopamine release likely involves retinal inputs to the SC, which coordinates several motivated behaviors important for survival (Redgrave and Gurney, 2006; Redgrave et al., 2010; Basso and May, 2017).

Recently, it has been proposed that ipRGCs can influence mesolimbic dopamine circuits via their excitatory projections to the hypothalamic preoptic area (Zhang et al., 2021). In this work, activation of ipRGCs or the corticotropin-releasing hormone-positive, GABAergic neurons in the preoptic area (POA) that they innervate promoted non-REM sleep, possibly by inhibiting the wakefulness-promoting VTA (Zhang et al., 2021). ipRGCs innervate the medial POA (mPOA) (Zhang et al., 2021; Santana et al., 2023) and lateral POA (lPOA) (Hattar et al., 2006; Li and Schmidt, 2018; Aranda and Schmidt, 2021; Santana et al., 2023), and both structures regulate motivated behaviors via glutamatergic (Gordon-Fennell et al., 2020; Tao et al., 2024) and GABAergic (McHenry et al., 2017; Gordon-Fennell et al., 2020) projections to the VTA. Thus, it is plausible that ipRGCs could contribute to the dopaminergic response to light via hypothalamic intermediates. Future work will be needed to validate this hypothesized connection, as published studies suggest that GABAergic projections from the POA promotes NAc dopamine release via disinhibition of VTA dopamine neurons (McHenry et al., 2017; Gordon-Fennell et al., 2019; Tao et al., 2024), although the local control of VTA microcircuits by lPOA inputs is complex and not fully understood (Gordon-Fennell et al., 2020). Because VTA projections can encode different types of sensory information depending on their downstream target (de Jong et al., 2018), it will be important to further explore regional heterogeneity in the ability of dopamine release to encode information about visual stimuli across the striatum, as well as identify upstream circuits that mediate these differences.

At this time, the ethological importance of the finding that LNAc encodes information about rapid, environmental lighting transitions is unknown but could represent a general saliency signal in nocturnal rodents that forage in the dark to increase alertness or prime a future action. Dopaminergic neurotransmission has been shown to serve various physiological functions depending on the release site and behavioral context, such as conveying stimulus value (Schultz et al., 2015; Berke, 2018); indicating when an outcome violates an expectation, such as in temporal difference reward prediction error models (Schultz, 1998; Keiflin and Janak, 2015; Watabe-Uchida et al., 2017); facilitating latent inhibition (Young et al., 2005; Kutlu et al., 2022) or associative learning (Fadok et al., 2010; Darvas et al., 2011; Jeong et al., 2022); promoting movement or action selection (Yin et al., 2008; Graybiel and Grafton, 2015; Coddington and Dudman, 2019); modulating motivational drive (Berridge and Robinson, 2016; Salamone and Correa, 2024); etc. Recently, we found that dopamine released in the NAc medial shell, but not in the LNAc, encodes information about future defensive actions, whereby the dopamine evoked by the appearance of threatening looming discs that mimic predator approach from above predicts the timing and vigor of the subsequent escape behavior (Fisher et al., 2025). In contrast, looming disc-evoked LNAc dopamine release did not reflect the saliency of a visual stimulus or movement kinematics. In fact, there was no detectable LNAc dLight1 transient at the onset of locomotion before or during threat presentation. Thus, additional research will be needed to determine how luxotonic dopamine responses in the LNAc influence visually guided animal behavior and relate to established theories of dopamine function.

In summary, we found that melanopsin loss subtly perturbs the mesolimbic response to light, which encodes information about the magnitude of environmental luminance changes. While this work contributes to the existing body of research delineating the functional significance of melanopsin in the rodent brain, it is unclear to what extent our findings apply to human subjects. The current studies were performed in mice, which are nocturnal and, subsequently, have a different retinal photoreceptor composition than humans in order to facilitate visual perception in dim lighting conditions (Volland et al., 2015). Mice also have poorer visual acuity than humans (Priebe and McGee, 2014) due to a reduction in the degree of binocular vision, depth perception, and ability to distinguish different wavelengths (Peirson et al., 2018). However, recent studies have challenged whether these differences reduce the mouse’s ability to respond to their visual environment (Samonds et al., 2019; La Chioma et al., 2020; Johnson et al., 2021; Williams et al., 2021). Luxotonic mesolimbic responses to unconditioned light stimuli have not, to our knowledge, been demonstrated in humans, but there is evidence to suggest that striatal dopamine plays a role in visual perception and complex visual processing (Harris et al., 1990; Van Opstal et al., 2014; Tomasi et al., 2016). Thus, future research is needed to better elucidate the complex interplay between visual pathways and downstream dopaminergic circuits critical for behavioral reinforcement, attention, and motivational control in different mammalian species.

## Data Availability

The original contributions presented in the study are included in the article/Supplementary material; further inquiries can be directed to the corresponding author.

## Supplementary material

SUPPLEMENTARY FIGURE S1 Spectrometer characterization of the outputs of the LED light engine used to generate light stimuli. The figure shows the output of the band pass-filtered UV (360 nm/28 nm), blue (475 nm/28 nm), green (555 nm/28 nm), and red (635 nm/22 nm) LEDs that were used to generate light stimuli at 100%, 50%, 25%, 10%, and 1% of maximal output in arbitrary spectrometer units (a.u.). The near infrared (NIR) LED was not used in the current study.

SUPPLEMENTARY FIGURE S2 Extended data: Dopamine responses to 1.0 μW/cm^2^ light in the lateral nucleus accumbens in *Opn4* knockout and wildtype control mice. **(A)** Across all stimuli, the peak dLight1 response to a ten-second, 1.0 μW/cm^2^ light stimulus in *Opn4*^+/+^ (*shown in magenta*) and *Opn4^-/-^* (*shown in black*) mice was dependent on the wavelength of the stimulus but not the mouse genotype (*n*_+/+_ = 16, *n*_−/−_ = 13; two-way repeated measures ANOVA with Bonferroni *post hoc* tests; *F*_3,81_ = 7.73, *p*_wavelength_ = 0.0017; *F*_1,27_ = 2.79, *p*_genotype_ = 0.11; *F*_3,81_ = 0.68, *p*_wavelength x genotype_ = 0.57). *Post hoc* tests indicated that the amplitudes of the dLight1 transients evoked by UV, blue, and green light were larger than those evoked by red light stimuli regardless of genotype (*shown in gray*). **(B)** Across stimuli, the effect of wavelength on the time to peak light-evoked LNAc dopamine release was dependent on the mouse genotype (*n*_+/+_ = 16, *n*_−/−_ = 13; two-way repeated measures ANOVA with Bonferroni *post hoc* tests; *F*_3,81_ = 37.43, *p*_wavelength_ < 0.001; *F*_1,27_ = 4.02, *p*_genotype_ = 0.055; *F*_3,81_ = 2.80, *p*_wavelength x genotype_ = 0.045). *Post hoc* tests indicated that the time to peak dopamine release evoked by UV light was longer in *Opn4^-/-^* mice relative to *Opn4*^+/+^ littermates. Asterisks show statistically significant comparisons between wavelengths within *Opn4*^+/+^ (*magenta*) and *Opn4^-/-^* (*black*) mice. **(C)** Across all stimuli, the full width at half maximum amplitude (FWHM) of the light-evoked dLight1 transient was dependent on the wavelength of the stimulus but not mouse genotype (*n*_+/+_ = 16, *n*_−/−_ = 13; two-way repeated measures ANOVA with Bonferroni *post hoc* tests; *F*_3,81_ = 11.27, *p*_wavelength_ < 0.001; *F*_1,27_ = 0.73, *p*_genotype_ = 0.40; *F*_3,81_ = 1.48, *p*_wavelength x genotype_ = 0.23). *Post hoc* tests indicated that the FWHM of dopamine transients evoked by UV light was significantly greater than those evoked by blue and red light regardless of genotype (*gray*). In all panels, * indicates *p* < 0.05, ** indicates *p* < 0.01, and *** indicates *p* < 0.001.

SUPPLEMENTARY FIGURE S3 Extended data: Dopamine responses to 0.001 μW/cm^2^ light in the lateral nucleus accumbens in *Opn4* knockout and wildtype control mice. **(A)** Across all stimuli, the peak dLight1 response to a ten-second, 0.001 μW/cm^2^ light stimulus in *Opn4*^+/+^ (*shown in magenta*) and *Opn4^-/-^* (*shown in black*) mice was dependent on the wavelength of the stimulus but not the mouse genotype (*n*_+/+_ = 16, *n*_−/−_ = 13; two-way repeated measures ANOVA with Bonferroni *post hoc tests*; *F*_3,81_ = 51.9, *p*_wavelength_ < 0.001; *F*_1,27_ = 3.17, *p*_genotype_ = 0.086; *F*_3,81_ = 0.87, *p*_wavelength x genotype_ = 0.46). *Post hoc* tests indicated that the amplitudes of the dLight1 transients evoked by UV, blue, and green light were larger than those evoked by red light stimuli, and the response to UV light was smaller than the response to blue light stimuli regardless of genotype (*shown in gray*). **(B)** Across all stimuli, significant main effects of wavelength and genotype on the time to peak light-evoked LNAc dopamine release was observed, but these factors did not interact (*n*_+/+_ = 16, *n*_−/−_ = 13; two-way repeated measures ANOVA with Bonferroni *post hoc* tests; *F*_3,81_ = 62.9, *p*_wavelength_ < 0.001; *F*_1,27_ = 7.81, *p*_genotype_ = 0.0095; *F*_3,81_ = 2.24, *p*_wavelength x genotype_ = 0.090). *Post hoc* tests indicated that the time to peak dopamine release evoked by red light was longer than those evoked by UV, blue, and green light regardless of genotype (*gray*). **(C)** Across all stimuli, the full width at half maximum amplitude (FWHM) of the light-evoked dLight1 transient was dependent on the wavelength of the stimulus but not mouse genotype (*n*_+/+_ = 16, *n*_−/−_ = 13; two-way repeated measures ANOVA with Bonferroni *post hoc* tests; *F*_3,81_ = 14.7, *p*_wavelength_ < 0.001; *F*_1,27_ = 0.034, *p*_genotype_ = 0.86; *F*_3,81_ = 0.13, *p*_wavelength x genotype_ = 0.94). *Post hoc* tests indicated that the FWHM of dopamine transients evoked by red light was significantly lower than those evoked by UV, blue, and green light regardless of genotype (*gray*). In all panels, * indicates *p* < 0.05, ** indicates *p* < 0.01, and *** indicates *p* < 0.001.

SUPPLEMENTARY FIGURE S4 Extended data: Dopamine responses to 0.0001 μW/cm^2^ light in the lateral nucleus accumbens in *Opn4* knockout and wildtype control mice. **(A)** Across all stimuli, the peak dLight1 response to a ten-second, 0.0001 μW/cm^2^ light stimulus in *Opn4*^+/+^ (*shown in magenta*) and *Opn4^-/-^* (*shown in black*) mice was dependent on the wavelength of the stimulus but not the mouse genotype (*n*_+/+_ = 8, *n*_−/−_ = 8; two-way repeated measures ANOVA with Bonferroni *post hoc tests*; *F*_3,42_ = 26.9, *p*_wavelength_ < 0.001; *F*_1,14_ = 3.10, *p*_genotype_ = 0.10; *F*_3,42_ = 1.25, *p*_wavelength x genotype_ = 0.30). *Post hoc* tests indicated that the amplitudes of the dLight1 transients evoked by UV, blue, and green light were larger than those evoked by red light stimuli, and the response to UV light was smaller than the response to blue light stimuli regardless of genotype (*shown in gray*). **(B,C)** Because no light-evoked dopamine transient was observed for 0.0001 μW/cm^2^ red light stimuli, the full width at half maximum amplitude (FWHM) and peak latency could not be calculated for this stimulus color and are omitted from figure panels. **(B)** Across all stimuli, the time to peak light-evoked LNAc dopamine release was dependent on the mouse genotype but not wavelength (*n*_+/+_ = 8, *n*_−/−_ = 8; two-way repeated measures ANOVA with Bonferroni *post hoc* tests; *F*_2,28_ = 0.85, *p*_wavelength_ = 0.44; *F*_1,14_ = 6.16, *p*_genotype_ = 0.026; *F*_2,28_ = 0.15, p_wavelength x genotype_ = 0.86). **(C)** Across all stimuli, FWHM of the light-evoked dLight1 transient was dependent on the wavelength of the stimulus but not mouse genotype (*n*_+/+_ = 8, *n*_−/−_ = 8; two-way repeated measures ANOVA with Bonferroni *post hoc* tests; *F*_2,28_ = 4.15, *p*_wavelength_ = 0.029; *F*_1,14_ = 0.22, *p*_genotype_ = 0.65; *F*_2,28_ = 0.87, *p*_wavelength x genotype_ = 0.43). *Post hoc* testing did not identify any significant differences in FWHM between individual wavelengths regardless of genotype. In all panels, * indicates *p* < 0.05, ** indicates *p* < 0.01, and *** indicates *p* < 0.001.
