# Supplementary figures and images for: Examining the role of the photopigment melanopsin in the striatal dopamine response to light
Source: Front Syst Neurosci. 2025 Apr 2;19:1568878. doi: 10.3389/fnsys.2025.1568878 (PMC12000111; doi:10.3389/fnsys.2025.1568878)

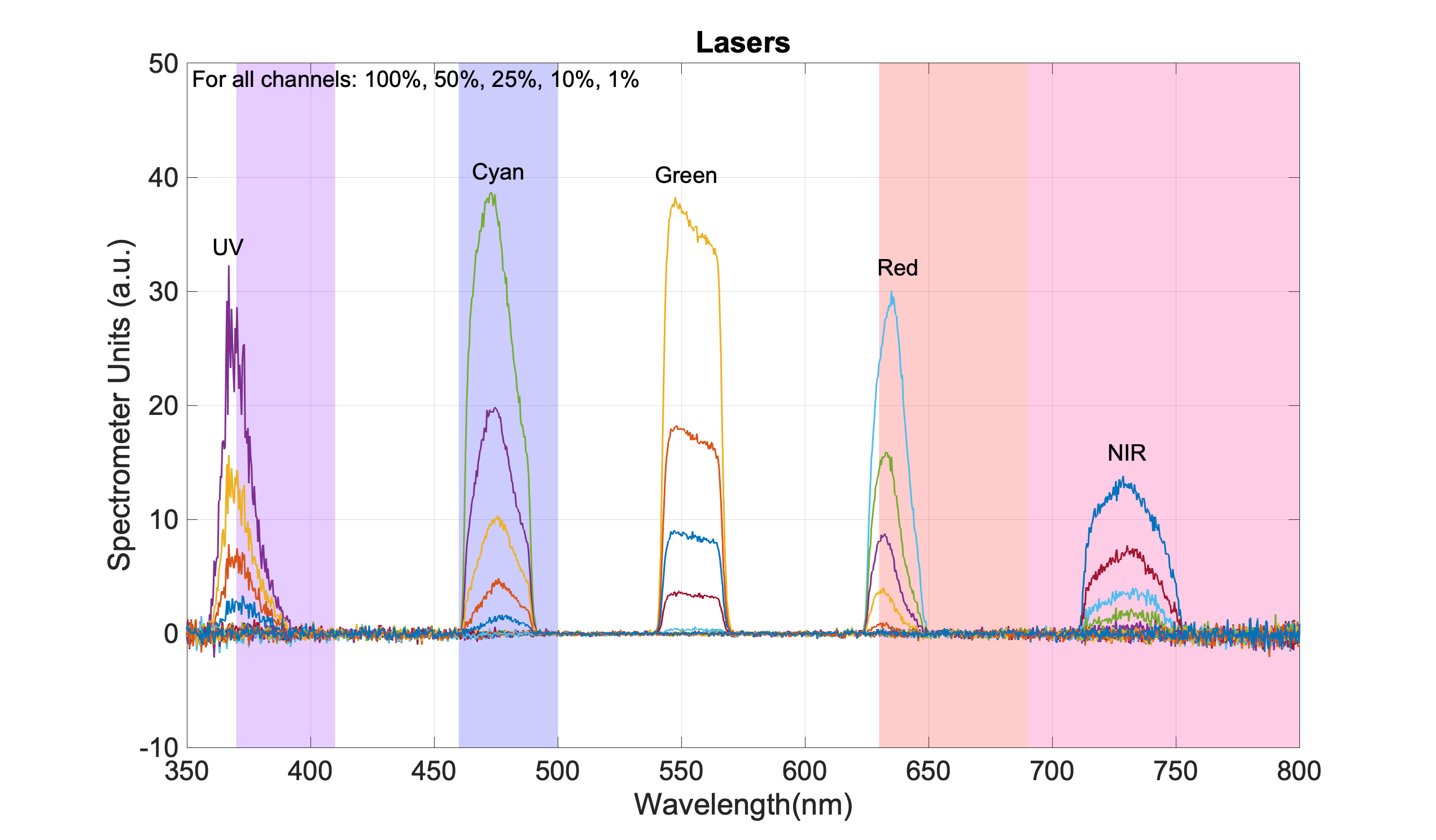

Supplement: SUPPLEMENTARY FIGURE S1 — Spectrometer characterization of the outputs of the LED light engine used to generate light stimuli. The figure shows the output of the band pass-filtered UV (360 nm/28 nm), blue (475 nm/28 nm), green (555 nm/28 nm), and red (635 nm/22 nm) LEDs that were used to generate light stimuli at 100%, 50%, 25%, 10%, and 1% of maximal output in arbitrary spectrometer units (a.u.). The near infrared (NIR) LED was not used in the current study. [file Image_1.PNG]

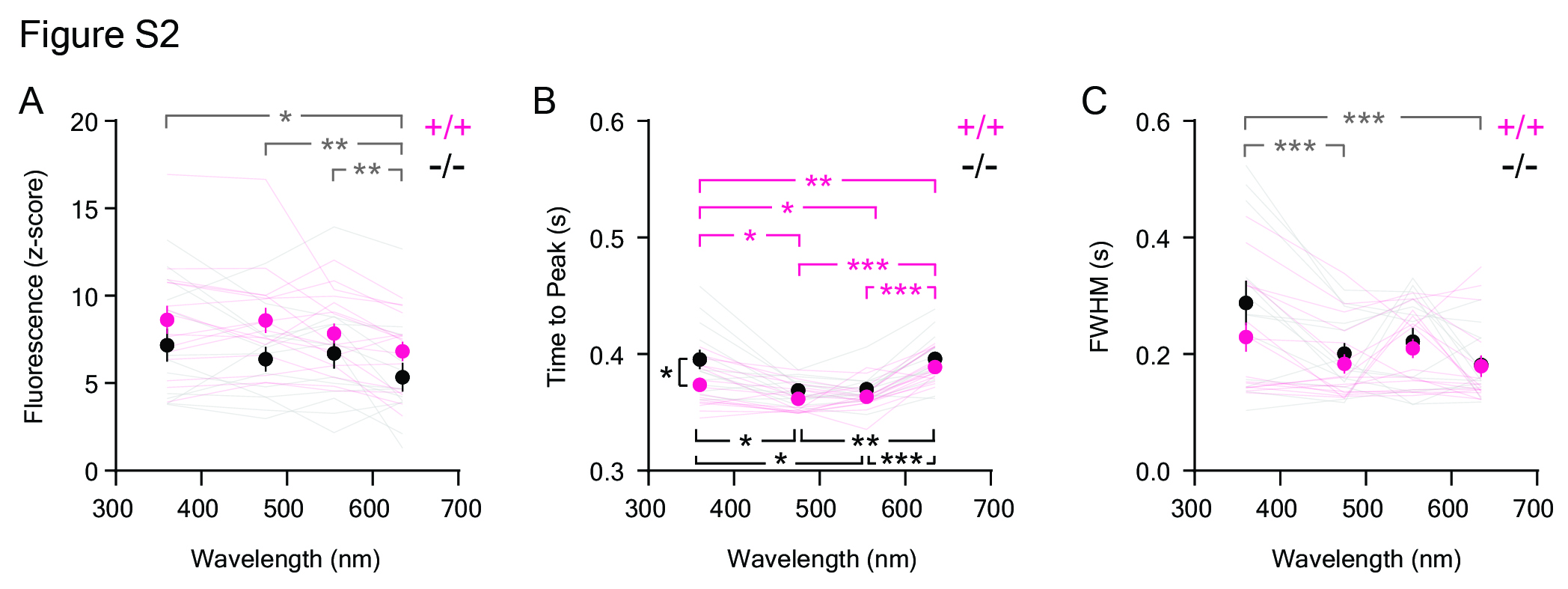

Supplement: SUPPLEMENTARY FIGURE S2 — Extended data: Dopamine responses to 1.0 μW/cm2 light in the lateral nucleus accumbens in Opn4 knockout and wildtype control mice. (A) Across all stimuli, the peak dLight1 response to a ten-second, 1.0 μW/cm2 light stimulus in Opn4+/+ (shown in magenta) and Opn4-/- (shown in black) mice was dependent on the wavelength of the stimulus but not the mouse genotype (n+/+ = 16, n−/− = 13; two-way repeated measures ANOVA with Bonferroni post hoc tests; F3,81 = 7.73, pwavelength = 0.0017; F1,27 = 2.79, pgenotype = 0.11; F3,81 = 0.68, pwavelength x genotype = 0.57). Post hoc tests indicated that the amplitudes of the dLight1 transients evoked by UV, blue, and green light were larger than those evoked by red light stimuli regardless of genotype (shown in gray). (B) Across stimuli, the effect of wavelength on the time to peak light-evoked LNAc dopamine release was dependent on the mouse genotype (n+/+ = 16, n−/− = 13; two-way repeated measures ANOVA with Bonferroni post hoc tests; F3,81 = 37.43, pwavelength < 0.001; F1,27 = 4.02, pgenotype = 0.055; F3,81 = 2.80, pwavelength x genotype = 0.045). Post hoc tests indicated that the time to peak dopamine release evoked by UV light was longer in Opn4-/- mice relative to Opn4+/+ littermates. Asterisks show statistically significant comparisons between wavelengths within Opn4+/+ (magenta) and Opn4-/- (black) mice. (C) Across all stimuli, the full width at half maximum amplitude (FWHM) of the light-evoked dLight1 transient was dependent on the wavelength of the stimulus but not mouse genotype (n+/+ = 16, n−/− = 13; two-way repeated measures ANOVA with Bonferroni post hoc tests; F3,81 = 11.27, pwavelength < 0.001; F1,27 = 0.73, pgenotype = 0.40; F3,81 = 1.48, pwavelength x genotype = 0.23). Post hoc tests indicated that the FWHM of dopamine transients evoked by UV light was significantly greater than those evoked by blue and red light regardless of genotype (gray). In all panels, * indicates p < 0.05, ** indicates p < 0.01, and *** indi [file Image_2.jpg]

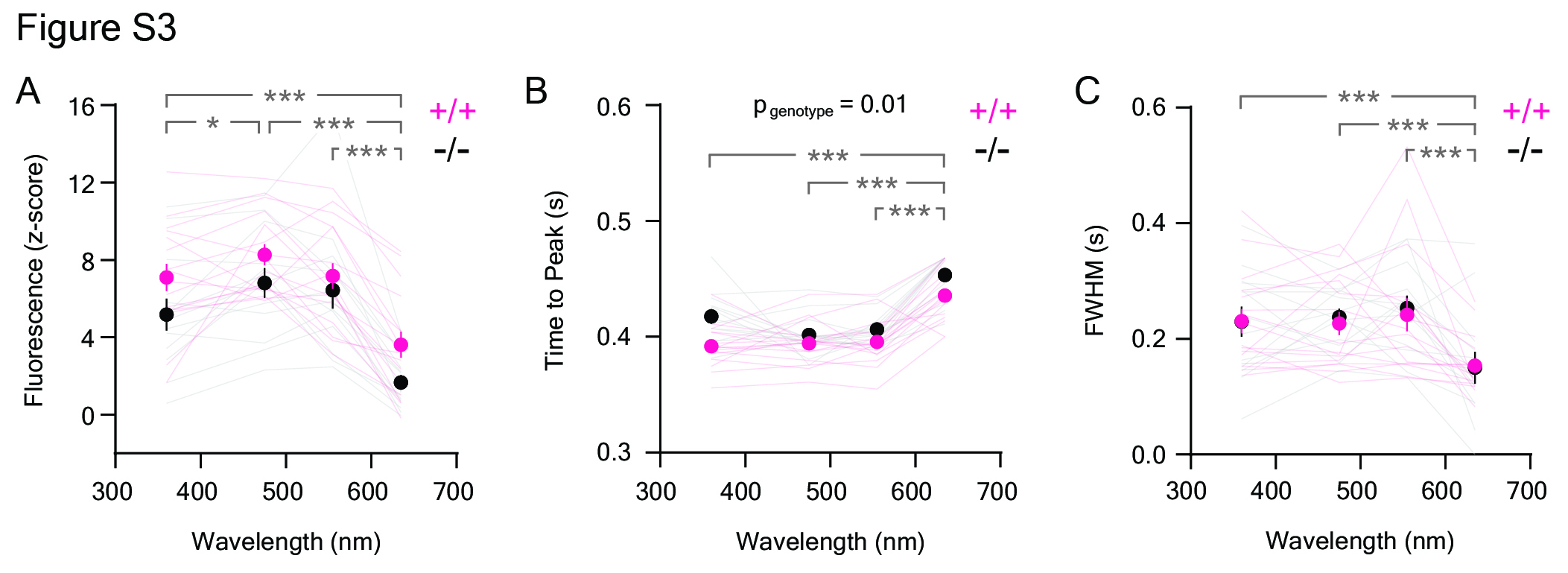

Supplement: SUPPLEMENTARY FIGURE S3 — Extended data: Dopamine responses to 0.001 μW/cm2 light in the lateral nucleus accumbens in Opn4 knockout and wildtype control mice. (A) Across all stimuli, the peak dLight1 response to a ten-second, 0.001 μW/cm2 light stimulus in Opn4+/+ (shown in magenta) and Opn4-/- (shown in black) mice was dependent on the wavelength of the stimulus but not the mouse genotype (n+/+ = 16, n−/− = 13; two-way repeated measures ANOVA with Bonferroni post hoc tests; F3,81 = 51.9, pwavelength < 0.001; F1,27 = 3.17, pgenotype = 0.086; F3,81 = 0.87, pwavelength x genotype = 0.46). Post hoc tests indicated that the amplitudes of the dLight1 transients evoked by UV, blue, and green light were larger than those evoked by red light stimuli, and the response to UV light was smaller than the response to blue light stimuli regardless of genotype (shown in gray). (B) Across all stimuli, significant main effects of wavelength and genotype on the time to peak light-evoked LNAc dopamine release was observed, but these factors did not interact (n+/+ = 16, n−/− = 13; two-way repeated measures ANOVA with Bonferroni post hoc tests; F3,81 = 62.9, pwavelength < 0.001; F1,27 = 7.81, pgenotype = 0.0095; F3,81 = 2.24, pwavelength x genotype = 0.090). Post hoc tests indicated that the time to peak dopamine release evoked by red light was longer than those evoked by UV, blue, and green light regardless of genotype (gray). (C) Across all stimuli, the full width at half maximum amplitude (FWHM) of the light-evoked dLight1 transient was dependent on the wavelength of the stimulus but not mouse genotype (n+/+ = 16, n−/− = 13; two-way repeated measures ANOVA with Bonferroni post hoc tests; F3,81 = 14.7, pwavelength < 0.001; F1,27 = 0.034, pgenotype = 0.86; F3,81 = 0.13, pwavelength x genotype = 0.94). Post hoc tests indicated that the FWHM of dopamine transients evoked by red light was significantly lower than those evoked by UV, blue, and green light regardless of genotype (gray). In all panels, * indicates p < [file Image_3.jpg]

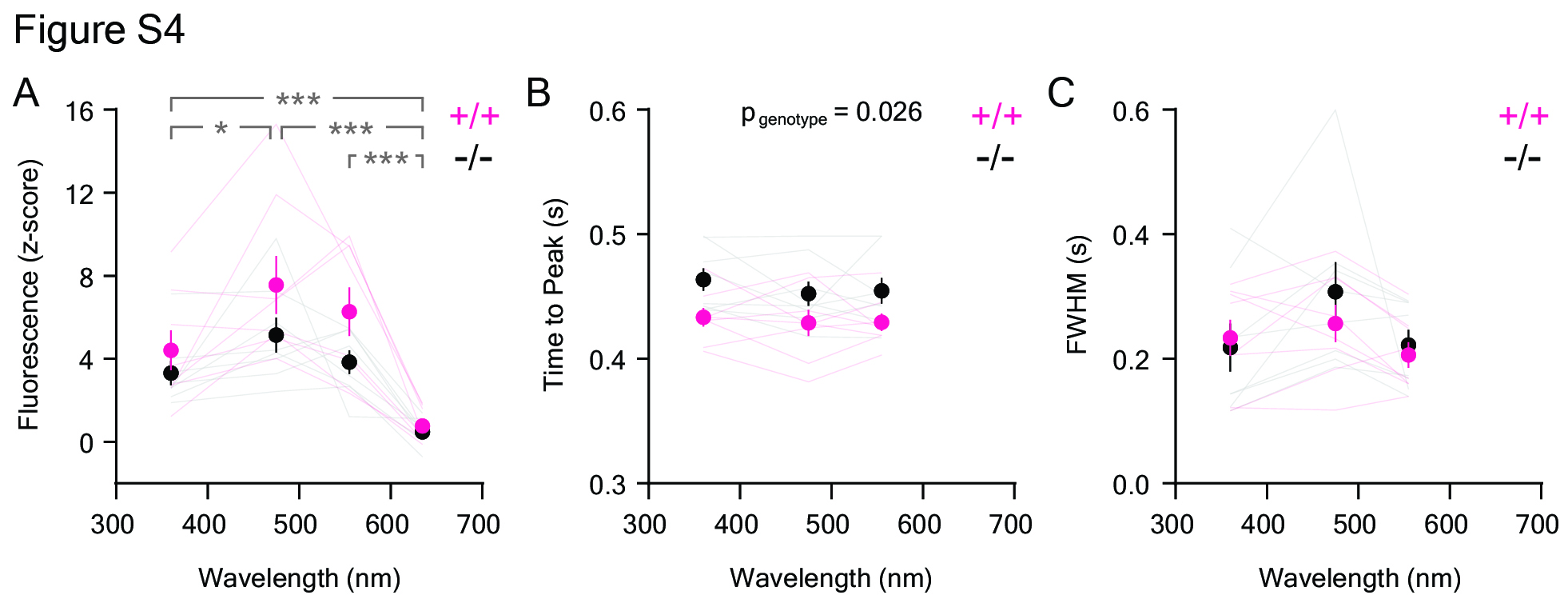

Supplement: SUPPLEMENTARY FIGURE S4 — Extended data: Dopamine responses to 0.0001 μW/cm2 light in the lateral nucleus accumbens in Opn4 knockout and wildtype control mice. (A) Across all stimuli, the peak dLight1 response to a ten-second, 0.0001 μW/cm2 light stimulus in Opn4+/+ (shown in magenta) and Opn4-/- (shown in black) mice was dependent on the wavelength of the stimulus but not the mouse genotype (n+/+ = 8, n−/− = 8; two-way repeated measures ANOVA with Bonferroni post hoc tests; F3,42 = 26.9, pwavelength < 0.001; F1,14 = 3.10, pgenotype = 0.10; F3,42 = 1.25, pwavelength x genotype = 0.30). Post hoc tests indicated that the amplitudes of the dLight1 transients evoked by UV, blue, and green light were larger than those evoked by red light stimuli, and the response to UV light was smaller than the response to blue light stimuli regardless of genotype (shown in gray). (B,C) Because no light-evoked dopamine transient was observed for 0.0001 μW/cm2 red light stimuli, the full width at half maximum amplitude (FWHM) and peak latency could not be calculated for this stimulus color and are omitted from figure panels. (B) Across all stimuli, the time to peak light-evoked LNAc dopamine release was dependent on the mouse genotype but not wavelength (n+/+ = 8, n−/− = 8; two-way repeated measures ANOVA with Bonferroni post hoc tests; F2,28 = 0.85, pwavelength = 0.44; F1,14 = 6.16, pgenotype = 0.026; F2,28 = 0.15, pwavelength x genotype = 0.86). (C) Across all stimuli, FWHM of the light-evoked dLight1 transient was dependent on the wavelength of the stimulus but not mouse genotype (n+/+ = 8, n−/− = 8; two-way repeated measures ANOVA with Bonferroni post hoc tests; F2,28 = 4.15, pwavelength = 0.029; F1,14 = 0.22, pgenotype = 0.65; F2,28 = 0.87, pwavelength x genotype = 0.43). Post hoc testing did not identify any significant differences in FWHM between individual wavelengths regardless of genotype. In all panels, * indicates p < 0.05, ** indicates p < 0.01, and *** indicates p < 0.001. [file Image_4.jpg]
